# Lignin Nanoparticles as A Promising Way for Enhancing Lignin Flame Retardant Effect in Polylactide

**DOI:** 10.3390/ma12132132

**Published:** 2019-07-02

**Authors:** Benjamin Chollet, José-Marie Lopez-Cuesta, Fouad Laoutid, Laurent Ferry

**Affiliations:** 1 Centre des Matériaux des Mines d’Ales, IMT Mines Ales, University of Montpellier, 30319 Alès, France; 2 Laboratory of Polymeric & Composite Materials, Materia Nova Research Center, 3 avenue Nicolas Copernic, B-7000 Mons, Belgium

**Keywords:** lignin nanoparticles, flame retardancy, polylactide, phosphorylation, biobased materials

## Abstract

The present study investigates the effect of using lignin at nanoscale as new flame-retardant additive for polylactide (PLA). Lignin nanoparticles (LNP) were prepared from Kraft lignin microparticles (LMP) through a dissolution-precipitation process. Both micro and nano lignins were functionalized using diethyl chlorophosphate (LMP-diEtP and LNP-diEtP, respectively) and diethyl (2-(triethoxysilyl)ethyl) phosphonate (LMP-SiP and LNP-SiP, respectively) to enhance their flame-retardant effect in PLA. From the use of inductively coupled plasma (ICP) spectrometry, it can be considered that a large amount of phosphorus has been grafted onto the nanoparticles. It has been previously shown that blending lignin with PLA induces degradation of the polymer matrix. However, phosphorylated lignin nanoparticles seem to limit PLA degradation during melt processing and the nanocomposites were shown to be relatively thermally stable. Cone calorimeter tests revealed that the incorporation of untreated lignin, whatever its particle size, induced an increase in pHRR. Using phosphorylated lignin nanoparticles, especially those treated with diethyl (2-(triethoxysilyl)ethyl) phosphonate allows this negative effect to be overcome. Moreover, the pHRR is significantly reduced, even when only 5 wt% LNP-SiP is used.

## 1. Introduction

Biobased polymers have expanded significantly during the last decade. Many scientific studies have shown the ability to develop polymers from renewable resources (such as corn, wheat, sugar cane, etc.) in order to propose alternatives to fossil-based polymers, notably polylactic acid (PLA) [1,2]. Following the same trend, the development of green additives and fillers rouses more and more interest in order to reduce the environmental footprint of flame retarded polymers and particularly the biobased ones.

Studies about the development of fire retardants from biobased materials have been reviewed and widely discussed [3,4]. For example, the use of modified or unmodified polysaccharides (starch [5], chitosan [6]), proteins [7], or phenolic biomass [8] has been studied to improve the fire behavior of polymer materials. Among the phenolic biomass, lignin has been intensely studied [9,10,11] because of its abundance and its char forming ability that make it a good candidate for reducing the flammability of polymeric materials. Lignin can act as flame retardant for isotactic polypropylene at a relatively low incorporation rate (15 wt%) compared to the amount of fire retardant usually used in polypropylene [12]. Lignin may also be combined with some phosphate compounds and aluminum hydroxide to provide an increase in the thermal degradation temperature, the combustion time and the amount of residue from combustion of polypropylene. The compatibilization of lignin in acrylonitrile butadiene styrene has been investigated [13]. It has been shown that the incorporation of lignin can reduce the peak of heat release rate (pHRR), total heat release (THR), and mass loss rate during the combustion of acrylonitrile-butadiene styrene. The use of organosolv and Kraft lignins in PLA has been compared and the results demonstrated that blends properties depend on the nature of lignin employed [14]. Both lignins reduced the pHRR and the THR during cone calorimeter test owing to the formation of an insulating char layer at the surface of the sample during combustion. However, a decrease of the time to ignition (TTI) and the thermal stability of PLA was also observed.

At the same time, nano-technologies have allowed the improvement of the fire behavior of polymers owing to several processes such as the modification of the degradation pathway of the polymeric matrix as well as the modification of the rheological behavior. The thermal stability and flammability of poly(methyl methacrylate) (PMMA) containing organo-modified montmorillonite (OMMT) and metal oxide nanoparticles or both have been examined [15]. Thermal stability and flammability were improved with increasing amounts of oxide nanoparticles. The combination of oxide nanoparticles and organoclays induced a synergistic effect on the thermal stability and the fire performance: TTI was enhanced and THR was reduced. The combustion behavior of poly(ethylene-co-vinyl acetate) (EVA) composites containing modified organoclay has been studied and results showed that pHRR reduction was strongly affected by the nanoclay dispersion state [16]. All these studies highlight the superior flame-retardant effect of nanoparticles when properly dispersed in the polymeric matrix.

Only a few studies have associated both bio and nano-technologies for the development of flame retardants additives. The utility of this approach has been demonstrated especially when nanoparticles are associated with phosphorous compounds in the case of cellulose nanocrystals [17]. Lignin may be potentially appropriate for this approach since lignin nanoparticles can be easily obtained in different ways: Sonication [18], chemical modification [19], and precipitation [20,21]. However, lignin nanoparticles have never been used as flame retardants for polymeric materials. On the other hand, chemical functionalization of lignin with phosphorus-based molecules has been shown as an effective method for enhancing flame-retardant behavior [14,22,23]. Thus, the association of lignin nanoparticles with surface modification by grafting of phosphorous compounds is expected to entail a flame-retardant effect that could be effective at low levels of loading.

In this study, lignin nanoparticles (LNP) were prepared from Kraft lignin microparticles (LMP) by dissolution-precipitation. LMP and LNP were functionalized using diethyl chlorophosphate and diethyl (2-(triethoxysilyl)ethyl) phosphonate. All these lignins were incorporated into polylactic acid by melt blending. Thermal properties and fire behavior of the blends were determined using thermogravimetric analysis (TGA) and cone calorimeter experiments.

## 2. Materials and Methods

### 2.1. Materials

PLA resin (3052D) was purchased from NatureWorks (NatureWorks, Minnetonka, MN, USA). Kraft water soluble lignin with a low sulfonate content, diethyl chlorophosphate, and diethyl (2-(triethoxysilyl)ethyl) phosphonate were purchased from Sigma-Aldrich (Sigma-Aldrich, St Quentin Fallavier, France). Diethyl (2-(triethoxysilyl)ethyl) phosphonate was purchased from Specific Polymers (Specific Polymers, Castries, France). Absolute ethanol, ethylene glycol, and acetone were purchased from PanReac AppliChem (PanReac AppliChem, Darmstadt, Germany). Hydrochloric acid solution (35%) was purchased from Fisher Scientific (Fisher Scientific, Illkirch, France).

### 2.2. Lignin Nanoparticle Preparation

Lignin nanoparticles (LNP) were prepared from Kraft water-soluble lignin microparticles (LMP), by precipitation from ethylene glycol solution. A 4 wt% solution of lignin in ethylene glycol was prepared at 30 °C under stirring for 2 h. A 0.25M aqueous HCl solution was added at the rate of 2 drops per minute. After addition, the solution was stirred for another hour. Then, the solution was dialyzed against ethanol over 4 days in order to provoke non-solvent precipitation of lignin.

### 2.3. Lignin Treatment

**Figure 1:**
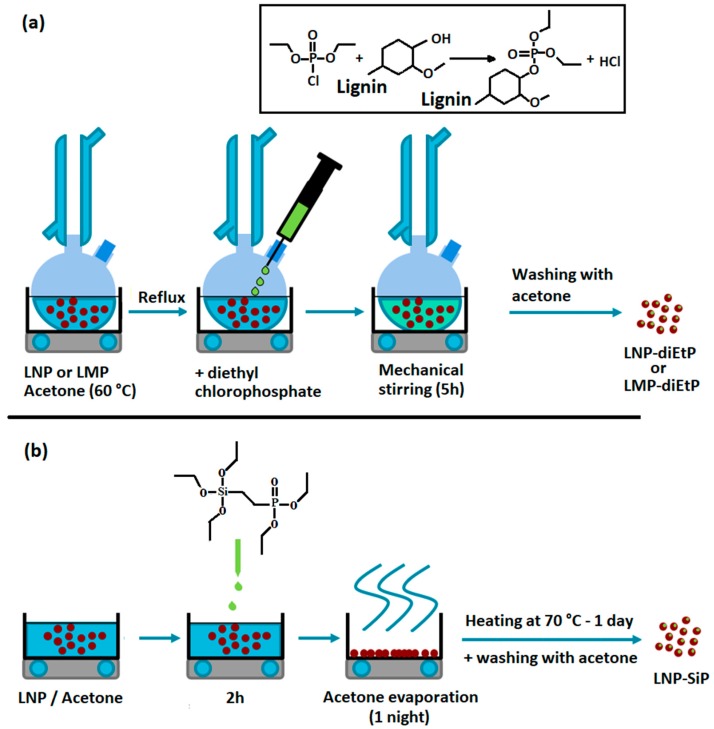
Schematic representation of lignin modification route used for grafting di-EtP (**a**) and SiP (**b**).

With diethyl chlorophosphate (diEtP) (Figure 1a): 30 g of lignin in 800 mL of acetone were introduced in a two-necked round-bottom flask equipped with a refrigerant and heated at 60 °C. At reflux, 15 g of diethyl chlorophosphate were slowly added and the mixture was stirred for 5 h. After cooling, solution was washed 3 times with acetone by centrifugation. Functionalized LMP and LNP were noted LMP-diEtP and LNP-diEtP.

With diethyl (2-(triethoxysilyl) ethyl) phosphonate (SiP) (Figure 1b): a mixture of 30 g of lignin and 100 mL of acetone were introduced in 500 mL one neck round-bottom flask under mechanical stirring. 15 g of silane agent was slowly introduced. The mixture was stirred over 2 h at room temperature and first dried under a ventilated hood over 1 day and at 70 °C for 1 night. The obtained dry powders have been washed 3 times with acetone by centrifugation. Functionalized LMP and LNP were noted LMP-SiP and LNP-SiP, respectively.

### 2.4. Composites Preparation

PLA 3052D containing 5 wt% and 10 wt% of the untreated and treated lignin micro and nano-particles were prepared in a Rheomix 3000 internal mixer from Thermo Fisher Scientific. PLA was first ground in 2 mm diameter powder and a suspension of lignin particles in acetone was added to the powder. Acetone was evaporated under a ventilated hood, then the powder mixture was dried overnight in a ventilated oven at 70 °C. Powders were blended in the internal mixer at 170 °C, first at 30 rpm during 3 min and then at 70 rpm during 7 min. The obtained composite was ground into 6 mm diameter pellets. Composite plates of 100 × 100 × 3 mm^3^ were compression molded from pellets using a Darragon thermopress at 170 °C. First pellets were brought into contact with the heated platens for 4 min, then a 50 bar pressure was applied for 2 min and finally a 100 bar pressure was applied for 2 more min. Plates were cooled in ambient atmosphere.

### 2.5. Lignin and Composites Characterization

a- Particle Size Analysis

Laser particle size analyzer LS 13 320 from Beckman-Coulter Company was used for determining particle size distribution of untreated and treated lignin nanoparticles in acetone. Particle size distribution was obtained by scattering of monochromatic light with a wavelength of 780 nm diffracted and transmitted through the suspension. Each analysis was at least reproduced 3 times and the maximum relative standard deviation for the median size was 10%. Quanta 200 FEG Scanning Electron Microscope (SEM) from FEI Company (FEI, Northeast Dawson, Hillsboro, OR, USA) was also used for investigating the lignin particles shape under high vacuum at a voltage of 12.5 kV and a working distance between 8.2 mm and 10.6 mm.

b- Phosphorus Content

Phosphorus content in functionalized lignins was determined using an Activa M Inductively Coupled Plasma (ICP) spectrometer from Horiba Jobin Yvon (HORIBA FRANCE SAS, Montpellier, France). In a first step, the organic matrix was decomposed by a mineralization process using HNO_3_ and H_2_SO_4_ solutions, followed by microwaves irradiation using a Milestone 1200-Mega from Gemini BV (Gemini BV, Apeldoorn, the Netherlands). A calibration curve was used to determine the exact amount of phosphorus in the samples.

c- Thermal Degradation

Thermal degradation of lignins and composites was studied by thermogravimetric analysis (TGA). Dried lignins samples and composites were submitted to a temperature ramp from 70 °C to 700 °C at a heating rate of 10 °C/min. TGA were performed under both air and nitrogen flow of 100 mL/min using a Setsys Evolution device from Setaram Instrumentation. 5%, 10%, and 50% weight loss temperatures (respectively T_5%_, T_10%_, and T_50%_) and char yield at 650 °C were determined.

d- Fire Properties

The fire behavior of the different composites was studied using a cone calorimeter from Fire Testing Technology. Samples of 100 × 100 × 3 mm^3^ were exposed to a 35 kW.m^−2^ radiant heat flux, corresponding to common heat flux in a mild fire scenario. Heat Release Rate (HRR) was measured as a function of time and Time To Ignition (TTI) and peak of Heat Release Rate (pHRR) were determined. For each sample, at least 2 tests were performed.

e- Degradation of PLA During Melt Processing

PLA thermal degradation during melt processing was investigated using a Steric Exclusion Chromatography (SEC) analysis. Samples were dissolved in chloroform (2 mg.mL^−1^) and filtered through a 0.2 μm filter. The molecular weight distributions were determined in CHCl_3_ at 23 °C using an Agilent size exclusion chromatograph equipped with a Knauer 2320 refractometer index detector and two PLGel columns (MIXED-D and 103 A). 20 μL of the sample solutions were injected into the columns using a flow rate of 1 mL/min. Monodisperse polystyrene standards (Polymer Laboratories Ltd., Church Stretton, UK) were used for the primary calibration. The number average molecular weight (Mn) was determined.

## 3. Results

### 3.1. Lignin Particles Structural and Thermal Properties

#### 3.1.1. Unmodified Lignin

**Figure 2:**
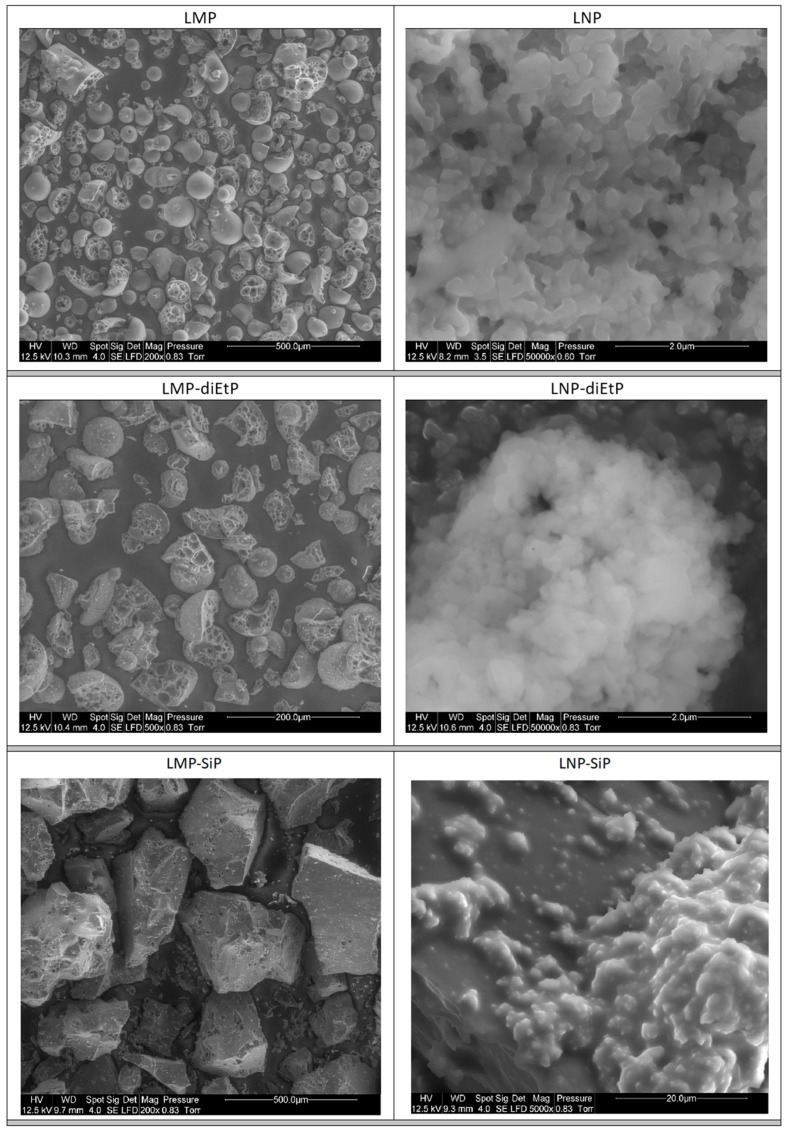
SEM micrographs of untreated and treated lignin microparticles (LMP) and lignin nanoparticles (LNP).

**Figure 3:**
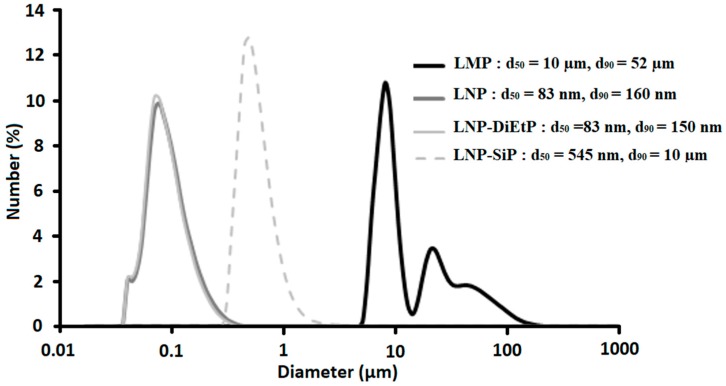
Number particle size distribution for LMP, LNP, and LNP-diEtP determined by laser particle size analyses in acetone.

First, the process of the lignin nanoparticles preparation was assessed. SEM observations (Figure 2) and particle size analyses (Figure 3) were used to evaluate the evolution of both particles size and their shape. It is important to notice that these analyses were performed on dry powders. Both laser particles size analyses and SEM observations evidenced that the original Kraft lignin contains microparticles with diameter ranging from 5 µm up to some hundred microns. Particles obtained using the dissolution-precipitation process exhibit different aspects. In fact, SEM micrographs highlight an important change of the powder morphology (Figure 2) that seems more like a porous and spongy material composed of the aggregation of small nanoparticles. Determination of particle size distribution supports this observation and shows an important change of the grain-size distribution curve that highlights the formation of very small nanoparticles with a d_50_ and d_90_ of only 80 nm and 160 nm, respectively.

**Figure 4:**
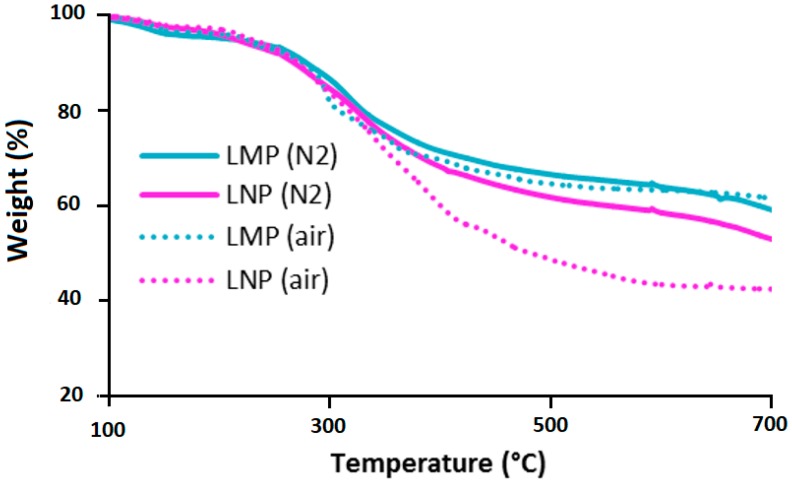
TGA curves, under air and nitrogen for LMP and LNP (10 °C/min).

Thermogravimetric analyses (TGA) were performed under both air and nitrogen to evaluate the effect of lignin particle size on its thermal stability (Figure 4). The thermal degradation of lignin is widely described in the literature [24]. Its decomposition starts by a water release followed by the first decomposition step (230–260 °C) that leads to the formation of low molecular weight products due to propanoid side chain cleavage. The main decomposition step occurs at higher temperature (250–450 °C) and leads to the production of a large quantity of methane due to the cleavage of lignin main chain and followed (above 500 °C) by several rearrangements and condensation reactions of the aromatic structure that leads to the formation of char structures, which decompose above 650 °C. This char is thermally stable only under anaerobic condition and decomposes totally in the presence of oxygen. TGA curves, under air and under nitrogen of LMP particles (Figure 4) follow the general decomposition steps described above and only the decomposition step occurring above 800 °C, corresponding to the thermo-oxidative decomposition of the char is affected by the nature of the atmosphere. In contrast, it is interesting to notice that the reduction of particle size strongly affect lignin thermo-oxidative degradation. Under air, the weight loss recorded above 350 °C is significantly more important in the case of LNP, while under pyrolytic conditions, only a slight difference between the curves of LMP and LNP was observed above 350 °C. This difference could not be attributed to lignin chemical structure since both particles were obtained from the same original kraft lignin and only particle size changed. The presence of oxygen played an important role for promoting this degradation. As LNP sample was composed of a porous material (aggregates of nanoparticles), the oxygen diffusion through this material during TGA is expected to be easier with respect to more compact micronic LMP particles. LMP were thus less exposed to oxygen since they present a lower specific surface area.

#### 3.1.2. Phosphorylated Lignin

The phosphorylation process is mainly used to increase the char stability during lignin aerobic thermal degradation and thus enhances the flame-retardant effect of lignin. First of all, the amount of grafted phosphorus with both diethyl chlorophosphate and diethyl (2-(triethoxysilyl) ethyl) phosphonate has been determined. ICP analyses (Table 1) highlight that, whatever the nature of grafting agent, phosphorus content is always higher when lignin nanoparticles are used, and the highest phosphorus content was obtained when lignin nanoparticles are treated with diethyl (2-(triethoxysilyl) ethyl) phosphonate (3.2%). Using multifunctional silyl-based phosphonate enables obtaining higher P content. This result was expected since diethyl chlorophosphate is able to react with only one lignin hydroxyl group. In contrast, SiP could establish 3 bonds with 3 hydroxyl groups provided by lignin or by self-condensation. However, it is important to notice that P content obtained is very low (only 0.1 wt%) when SiP is used with LMP. This result reflects the competition between grafting reaction on lignin and self-condensation. In fact, if only a few hydroxyl groups are present on the surface of lignin particles, the self-condensation between SiP molecules will consume the main part of the reactive functions of the modifying agent at the expense of grafting reactions. This result also highlights that the following cleaning procedure enables the elimination of ungrafted molecules.

However, grafting SiP induces some changes on the lignin particles structural and thermal properties. Firstly, in contrast to diEtP that does not affect lignin particle size, using SiP induces a significant increase in lignin particle size. Indeed, Figure 3 shows that lignin nanoparticles modified with diethyl chlorophosphate exhibit similar size compared to unmodified nanoparticles, while the size of lignin nanoparticles increases from 83 nm (d_50_) and 150 nm (d_90_) for LNP to 545 nm (d_50_) and 10 µm (d_90_) for LNP-SiP. The increase of lignin particle size observed in the case of LNP treated with SiP results from self-condensation of SiP as well as from the coupling of different nanoparticles due to the multifunctional reactivity of SiP. Such behavior has also been observed in the literature and resulted in an increase of lignin molecular weight [17] and lignin particle size [19]. SEM observations performed on dry LNP-SiP (Figure 2) support this result since the morphology of LNP-SiP particles changes and aggregates formed from small particles are evidenced. Moreover, EDX analysis of these aggregates reveals the presence of high concentrations of Si and P. It is important to notice that PLA / lignin nanocomposites were prepared by mixing PLA powders with a suspension of lignin nanoparticles into acetone in order to avoid the aggregation of lignin nanoparticles during the drying process. Hence, particle size analyses carried out in acetone provide a more accurate information upon the particle size and the particle size determined from SEM images is strongly dependent on the drying process that induces particle aggregation.

**Figure 5:**
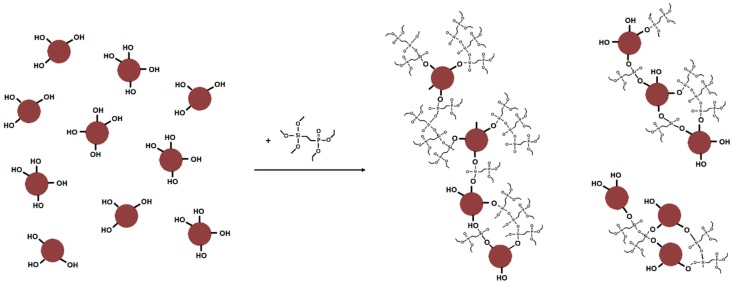
Schematic representation of LNP-SiP particles.

All these observations enable us to propose the following schematic representation of LNP-SiP (Figure 5) that explains the origin of the high P content as well as the significant increase of particles size in the case of LNP modified with SiP.

**Figure 6:**
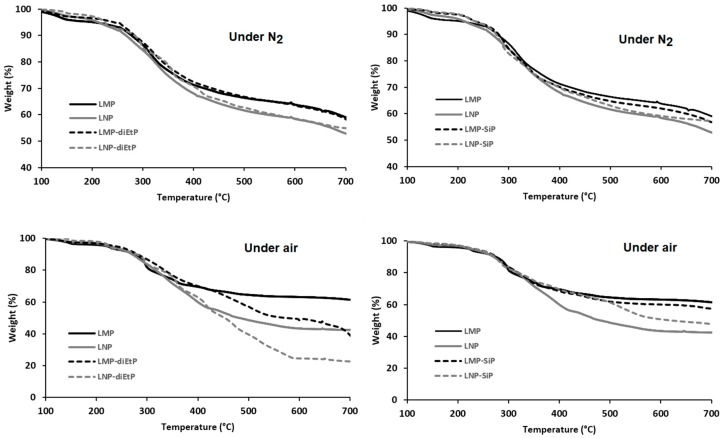
TGA curves of untreated and phosphorylated lignin particles (under air and nitrogen, 10 °C/min).

The effect of lignin phosphorylation on its thermal stability has been also evaluated using TGA analysis (Figure 6). The phosphorylation process of lignin is designed to increase the thermo-oxidative resistance of the char but mainly this reaction induces its premature thermal decomposition [10]. In our case, TGA analysis performed under nitrogen does not show any effect of the phosphorylation process on either the thermal stability or the amount of the final residue.

On the contrary, under air, the presence of phosphorus induces important changes in TGA curves (Figure 6) and the thermal behavior of modified lignins is significantly affected by the nature of the phosphorous agent used. In the case of lignin treated with diEtP, a significant reduction of the thermal stability of both modified lignin particles was observed above 400 °C. Instead of the plateau observed between 400 °C and 700 °C for both unmodified lignin particles, which corresponds to the formation of thermally stable char residues, the weight loss still occurs gradually above 400 °C in the case of LMP-diEtP and LNP-diEtP. It is worth to mention that the final residues at 700 °C are lower in the case of treated lignins, whatever their particle size. The effect of phosphorus is surprising since it was expected to act as a char promoter and not to induce char degradation. In the presence of SiP agent, the thermal behavior of treated and untreated LMP particles is not significantly affected. This result is due to the low content of grafted SiP. However, in the case of LNP, the effect of surface modification is more significant, and an important enhancement of the char thermal stability is observed above 400 °C. In fact, the degradation of the char previously observed with diEtP is strongly limited and the phosphorous agent acts mainly as a stabilizing agent. The amount of the final char at 700 °C is slightly higher for SiP treated LNP, but not in the case of treated microparticles.

### 3.2. Composites Thermal Degradation and Fire Behavior

It has been proven that lignin could be advantageously used to improve the char formation during the combustion of polymeric matrices [13,14,17]. However, all works reported in literature studied the effect of lignin microparticles and none of them concerned the effect of lignin nanoparticles. At least 20 wt% of lignin was considered, in order to significantly improve the composite fire behavior. Using lignin at nanoscale is expected to enable reducing its incorporation rate, while maintaining its flame-retardant effect owing to “the nano” effect [25]. In fact, generally speaking, nanoparticles, when correctly dispersed, are well known to reduce pHRR during cone calorimeter test.

#### 3.2.1. Effect of Untreated Lignin Content and Particle Size

**Figure 7:**
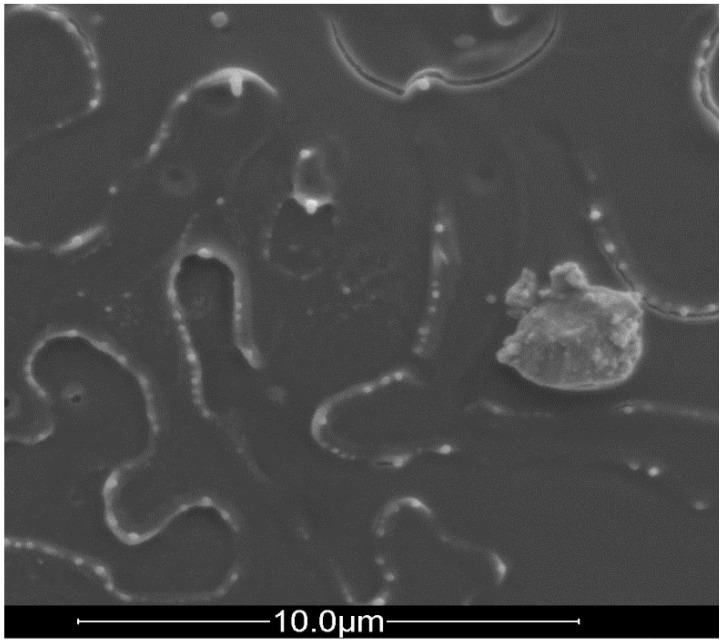
SEM image of PLA containing 5wt% LNP (White dots represent LNP).

First, we investigated the effect of lignin particle size on PLA degradation during melt processing, thermal stability by TGA and fire properties using cone calorimeter test. Both lignin micro and nano-particles were incorporated in PLA by melt mixing at different contents (5 and 10 wt%). SEM observation in Figure 7 evidences a rather good LNP dispersion since different nanoparticles of about 100 nm are observed as well as some aggregates of about 5 µm.

It has been reported in a previous work [17] that the incorporation of lignin into PLA presents some drawbacks: PLA degradation occurs during melt processing due to the presence of some degradant groups such as phenolic and carboxylic functions, as well as sulfur groups present at the surface of lignin particles.

**Figure 8:**
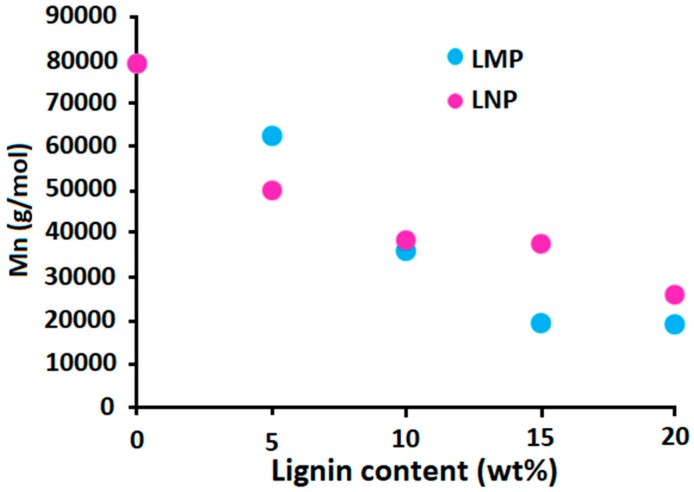
Effect of lignin size and content on the evolution of the number average molecular weight of processed PLA.

Thus, the effect of the incorporation of both lignins (nanoparticles and microparticles) on the thermal degradation of PLA during melt processing was first studied to evaluate whether the incorporation of very small lignin particles, which are supposed to exhibit higher specific surface area (1 m²/g for LMP and around 70 m²/g for LNP theoretically), induces higher degradation with respect to lignin microparticles. Figure 8 and Table 2 shows the evolution of the number average molecular weight (Mn) of processed PLA containing different lignin contents. The results indicate that increasing lignin content, whatever the particle size, induces significant reduction of Mn. The incorporation of 10 wt% lignin particles induces a reduction of about 50% of PLA molecular weight that decreases from 80,000 g/mol to 40,000 g/mol. Using 20 wt% LMP or LNP induces further reduction of Mn up to 20,000 g/mol. The PLA composite presenting such a low molecular weight will exhibit very low mechanical properties. To overcome this limitation, it is important to reduce the lignin content.

**Figure 9:**
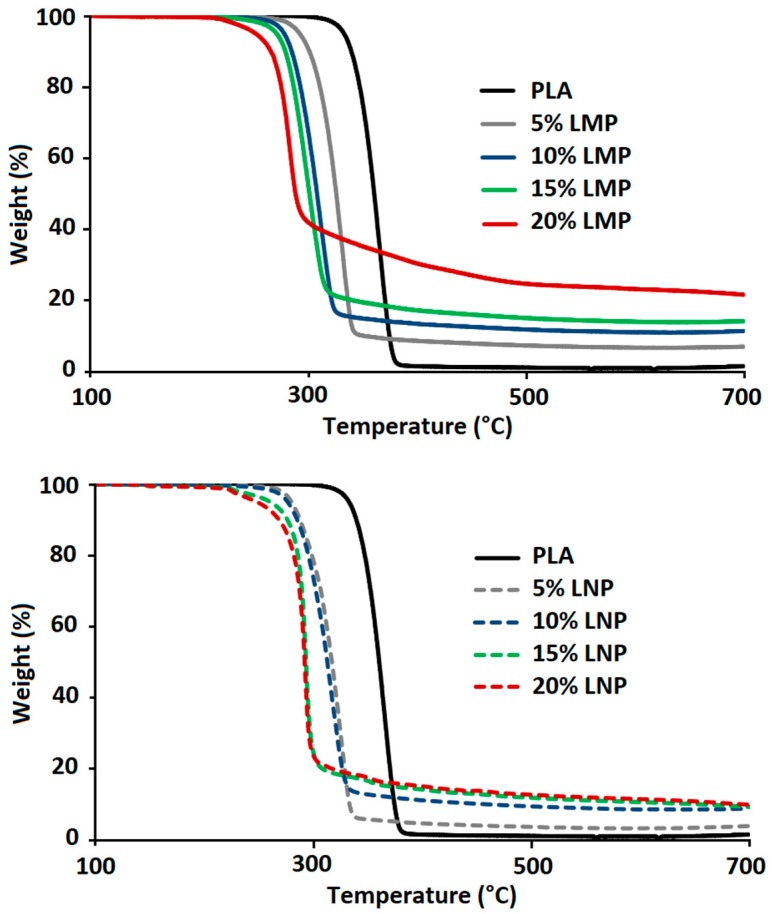
Effect of lignin size and content of PLA thermal stability during TGA under nitrogen.

Increasing lignin content, whatever the particle size, also induces an important reduction of PLA thermal stability during TGA analysis under nitrogen (Figure 9). Using lignin nanoparticles does not induce further degradation and the TGA curve of the composite containing 20 wt% lignin remains slightly similar whatever the particle size. Similar reduction of PLA thermal stability in the presence of lignin has been also evidenced for PLA containing 20 wt% lignin [14,26] and was attributed to the action of degradant lignin phenolic hydroxyl and carboxylic groups. Using lignin nanoparticles, that present higher surface area does not induce any further reduction of PLA thermal stability with respect to microparticles, since PLA degradation is induced by degradant groups formed during lignin thermal decomposition and not by those present at the surface. The latter are only effective at low temperature and mainly induce PLA degradation during melt processing.

**Figure 10:**
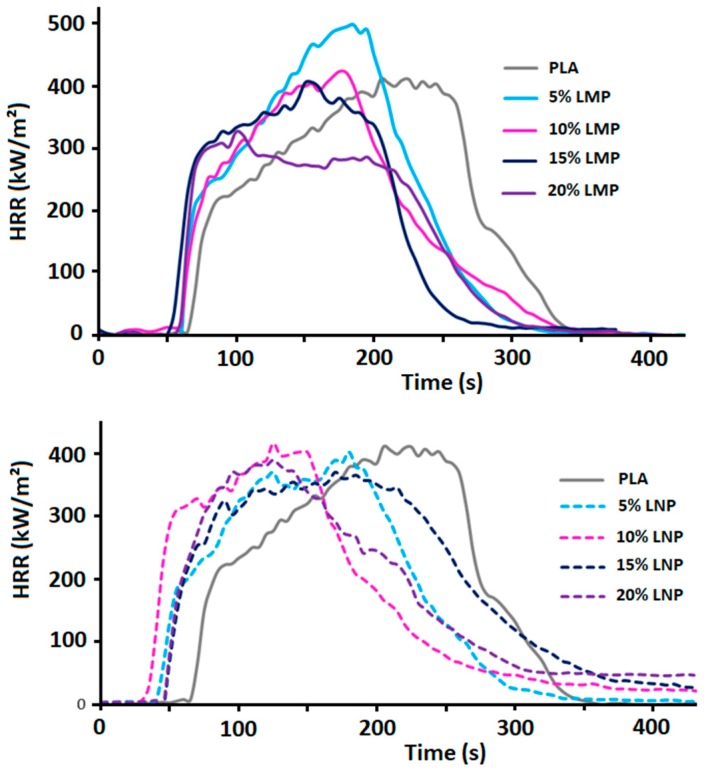
HRR curves of PLA containing different LMP and LNP content.

**Figure 11:**
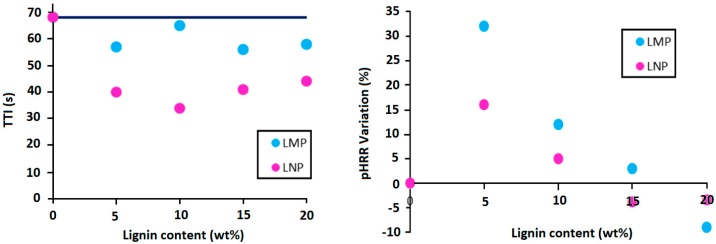
Evolution of time to ignition (TTI) and pHRR variation of PLA containing 5; 10; 15 and 20 wt% LMP and LNP.

The incorporation of untreated lignins entails some modifications of the fire behavior of PLA assessed using cone calorimeter test at 35 kW/m². Figure 10 shows pHRR curves of PLA containing different lignin contents and Figure 11 summarizes these results. In fact, both LMP and LNP induce important reduction of the resistance to ignition of PLA. Reduction of TTI in the presence of lignin has already been reported in the literature about PLA [26], PBS [22], and ABS [23]. It was attributed to several factors such as polymer thermal degradation, modification of composites emissivity, heat absorption and thermal conductivity. However, the reduction of PLA composites resistance to ignition is higher in the presence of nanoparticles and therefore lower TTI are observed (Figure 11). Using lignin nanoparticle thus induces important changes in physical and physico-chemical properties of the composite that significantly affects the composites resistance to ignition. The reason behind this effect is not clearly established but may be induced by an increase of nanocomposite heat absorption that could promote a quicker heating and the rapid formation of combustible volatile products. Further studies are needed for a better understanding of this effect.

Moreover, the incorporation of lignin particles, whatever their size, does not induce any significant reduction of pHRR except the composition containing 20 wt% LMP that only presents however a slight reduction of about 10%. In addition, using reduced lignin content does not present any pHRR reduction. Using such a low lignin content (5 wt%) is not enough to promote the formation of a continuous protective layer and only an ‘islands-in-the-sea’ structure is generated.

**Figure 12:**
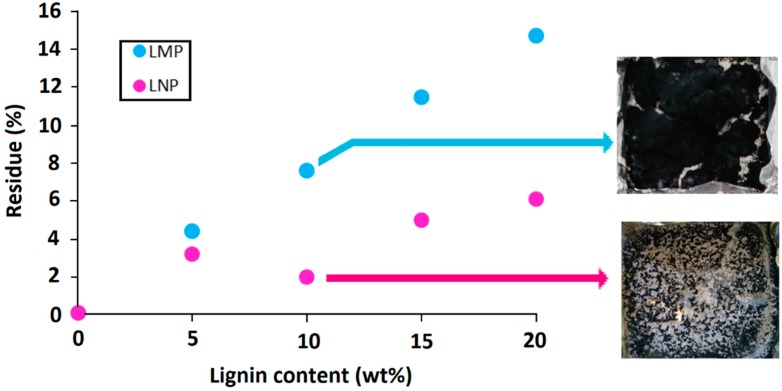
Effect of lignin size and content on the amount of char residue formed during cone calorimeter test.

The amount of the final char left after cone calorimeter test is mainly affected by lignin particle size (Figure 12). Using lignin nanoparticles induces the formation of lower char residue at the end of the test because of some incandescence phenomena that induce char degradation after the flame out.

Fire testing results clearly indicate that no superior effect was obtained using untreated lignin nanoparticles. Performances remain similar to those obtained with microparticles.

#### 3.2.2. Effect of Phosphorylated Lignin

The results obtained with untreated lignin nanoparticles did not highlight any superior effect of LNP with respect to LMP. The other way for taking advantage of lignin nanoparticles is to modify their surface by phosphorous based compound in order to limit their degradant effect, enhancing the amount of the char as well as its thermal stability for developing nanocomposites presenting high molecular weight and improved fire performance. With this view, untreated and phosphorylated lignin micro and nano particles were incorporated into PLA at reduced content (5 and 10 wt%). Their effect on PLA degradation, its thermal stability and fire behavior were studied.

Phosphorylation of lignin nanoparticles doesn’t enable significant increase of PLA thermal stability during melt processing. In fact, PLA molecular weights remain similar to those obtained with untreated LMP and LNP. Some changes are observed but do not demonstrate a significant effect and the Mn remains lower to 55,000 g/mol, while PLA containing 10 wt% untreated lignin microparticles and nanoparticles present Mn around 40,000 g/mol. The effect resulting from the presence of LNP-SiP is a little bit disappointing regarding the presence of a significant amount of non-degradant SiP groups at the surface of lignin. The number of average molecular weight of PLA composites containing LNP-SiP and LMP-SiP are very similar to those obtained with untreated LNP and LMP, respectively. The presence of SiP seems to not affect PLA thermal degradation during melt processing.

**Figure 13:**
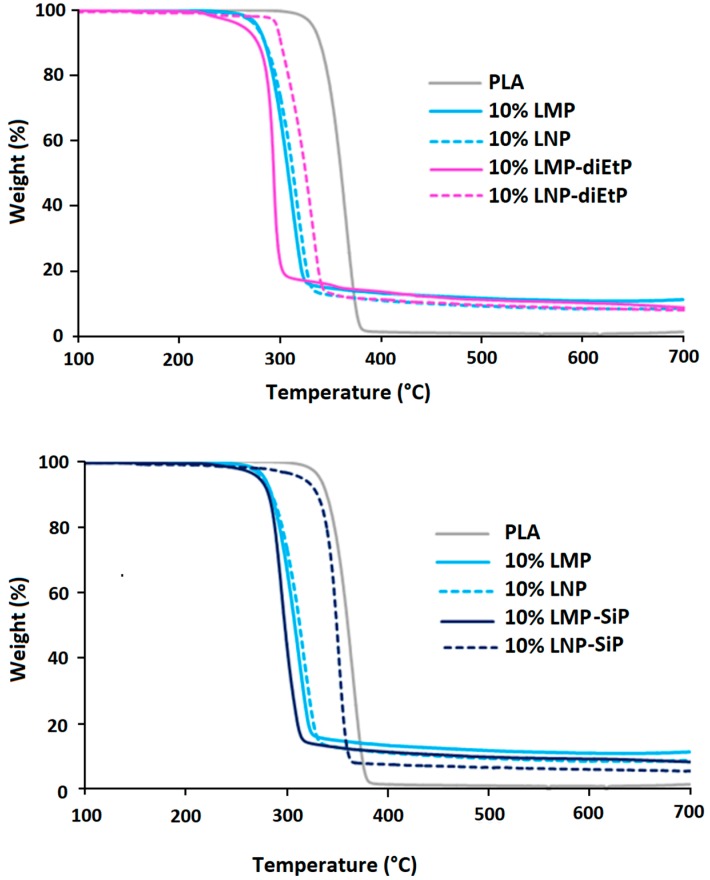
TGA curves of PLA and PLA composites under nitrogen.

SEC analysis enables determining the degradant effect of additives on PLA during melt processing at 170 °C while TGA analysis gives information on composites thermal stability at higher temperatures. TGA tests were run on dried samples, with a ramp of temperature from 100 °C to 700 °C. From these analyses, temperatures corresponding to 5% weight loss (T_5%_), 10% weight loss (T_10%_), 50% weight loss (T_50%_), as well as the value of the final char at 650 °C, were determined. TGA curves are presented in Figure 13 and data summarized in Table 3.

**Figure 14:**
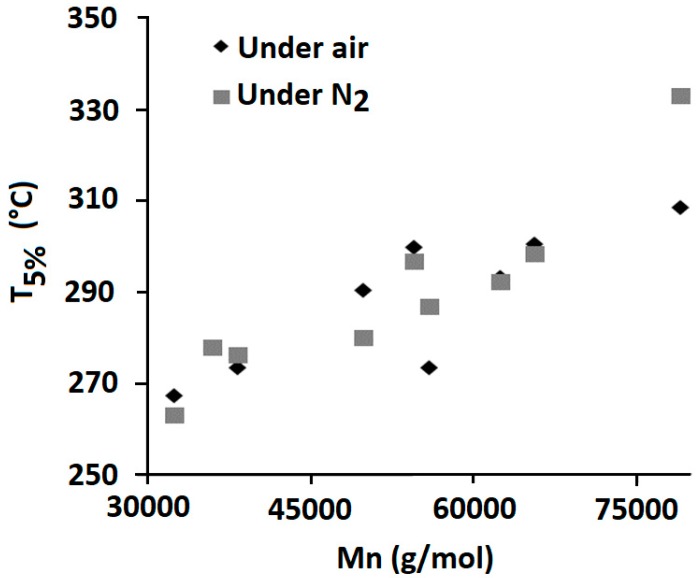
T5% as function of Mn.

The addition of untreated lignin, whatever its particle size, induced an important reduction of PLA thermal stability for all compositions. T_5%_ and T_10%_ were reduced, respectively, from 333 °C and 340 °C for neat PLA to below 290 °C and 300 °C in the presence of lignin particles. Following the same trend, T_50%_ was decreased from 360 °C to below 326 °C. Figure 14 shows that some correlation exists between T_5%_ and PLA chain molecular weight. The more Mn was reduced and the lower was T_5%_. This observation has already been reported in the literature [14]. As previously mentioned, this could be attributed to the degradant effect of some moieties present at the lignin surface and seems to indicate that the thermal degradation occurring during melt processing continued to occur at higher temperature during TGA experiments. Another hypothesis could be that thermal stability of PLA depends on the molecular weight of its macromolecules [27]. It means that shorter polymer chains would be more easily thermally degraded than larger polymer chains. It is worth mentioning that the use of nanoparticles did not induce any further degradation since T_5%_ and T_10%_ are similar to those obtained with 10 wt% LMP. These results showed that lignin particle size did not induce any significant modification of PLA thermal stability. Moreover, the incorporation of both lignins also induced the formation of some char during thermal decomposition under both air and nitrogen. However, this char was only thermally stable under anaerobic conditions. In fact, under air, almost no residue (maximum 1.3%) was left at the end of the test. At similar incorporation content, LMP enabled obtaining higher char yield than LNP. This could be due to the volatilization of a part of lignin nanoparticles with the degradation product. Moreover, under nitrogen, experimental char yields of the composites filled with LMP were higher than theoretical ones. This result evidences the protective effect of lignin that, owing to its char forming ability, avoids total PLA degradation, leading to a higher residue. With respect to untreated lignins, the incorporation of chemically treated lignin particles into PLA entailed different changes on composites thermal stability. While the presence of phosphorylated microparticles induced the early degradation of PLA under nitrogen, the incorporation of LNP-SiP nanoparticles enabled maintaining the thermal stability of PLA since the nanocomposite TGA curve was very close to that of pristine PLA. These results clearly indicate the interest of using phosphorylated nanoparticles instead of microparticles and especially those treated by SiP. The superior effect of LNP-SiP nanoparticles is related to their higher phosphorus content.

**Figure 15:**
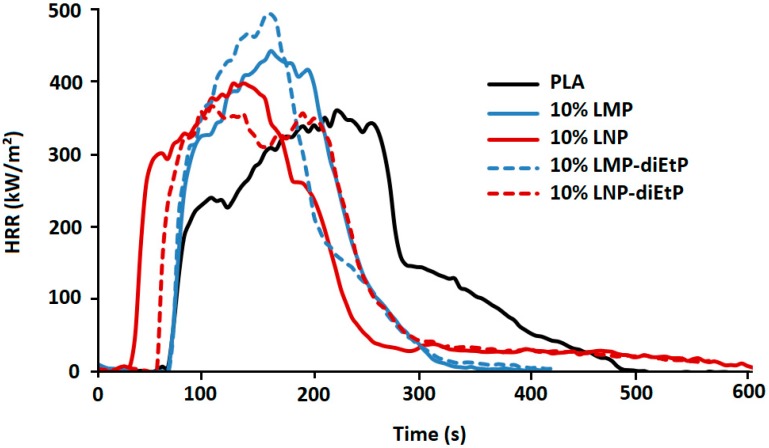
HRR curves of PLA during cone calorimeter test.

The effect of the incorporation of phosphorylated lignin nanoparticles on the composites fire behavior was investigated using cone calorimeter tests. As can be seen in Figure 15 and Table 4, the phosphorylation of lignin nanoparticles using diEtP enables limiting the reduction of TTI observed when untreated nanoparticles are used. Hence, time to ignition increases from 34 s obtained with 10 wt% LNP to 54 s when LNP-diEtP are used. This is likely due to the increase of the composite thermal stability due to the grafting of non-degradant diethylphosphate groups on the degradant hydroxyl groups, as was attested by both SEC and TGA analysis. However, using phosphorylated lignin with diEtP, whatever its particle size, does not enable any reduction of pHRR that remains at least similar to that of pristine PLA. The limited flame-retardant effect of these lignins seems to be mainly governed by the low phosphorus content grafted when diEtP is used.

**Figure 16:**
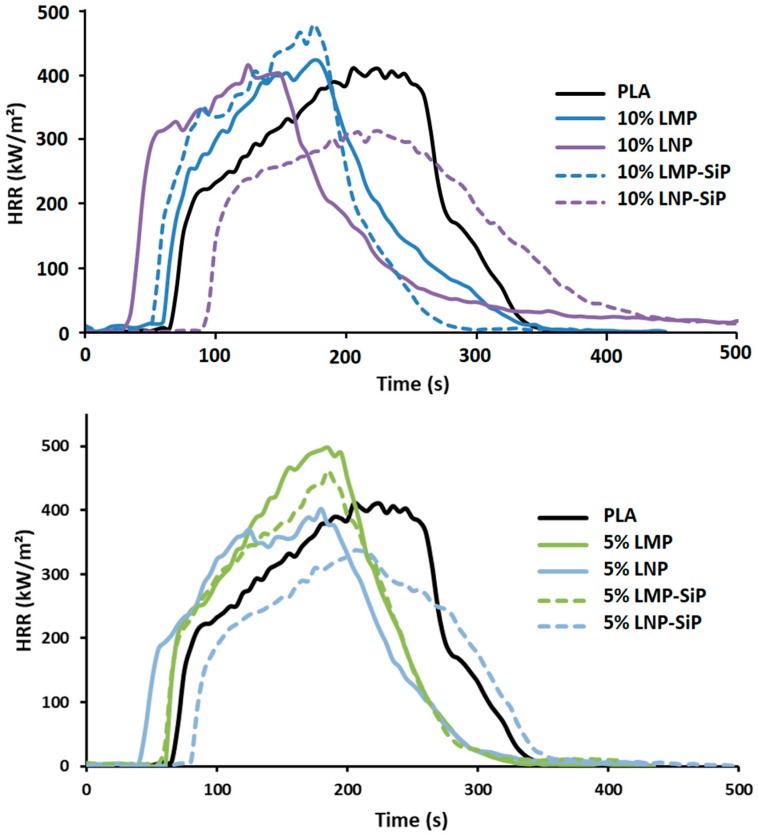
HRR curves of PLA during cone calorimeter test.

**Figure 17:**
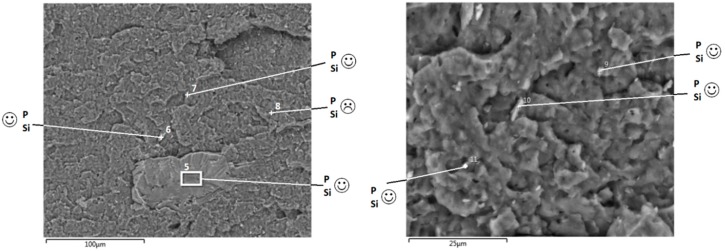
SEM observations and EDX analysis of PLA containing 10 wt% LNP-SiP.

However, using SiP as grafting agent enables obtaining higher performances with treated lignin nanoparticles (Figure 16). Hence, the incorporation of 10 wt% LNP-SiP entails an important increase of time to ignition that reaches 86 s and exceeds thus the time to ignition of pristine PLA. This result confirms the interest of using LNP-SiP that was shown to maintain the thermal stability of PLA as noted from TGA analysis. In addition, using 10 wt% LNP-SiP enables a significant reduction of pHRR of about 18%. This result is very important since this it is the first time that is reported that using so reduced lignin content (only 10 wt%) enables such significant flame-retardant effects, i.e., TTI increase and pHRR reduction. SEM observations and EDX analysis evidence a homogeneous dispersion of LNP-SiP in the composite (Figure 17). In fact, different EDX analyses were performed at different zones and most of them evidence the presence of both P and Si. It is important to notice that SEM analyses do not evidence the presence of numerous large particles aggregates in the composites, but only few of them are visible. The major LNP-SiP particles are properly dispersed.

Interestingly enough, the flame retardant effect of LNP-SiP remains effective even at more reduced content. Indeed, the incorporation of only 5 wt% LNP-SiP (Figure 16) still allows for obtaining high time to ignition (84 s) and reduced pHRR (−11%). Despite those noticeable effects, the amount of the final residue is low (1%). This result was assumed to be due to the incandescence phenomenon that induces char degradation after the flame out.

## 4. Conclusions

Lignin nanoparticles were synthesized from Kraft lignin. Both micro and nano lignins were functionalized with diethyl chlorophosphate and diethyl (2-(triethoxysilyl) ethyl) phosphonate. Using diethyl (2-(triethoxysilyl) ethyl) phosphonate enables grafting higher P content. Moreover, highest P content was obtained with lignin nanoparticles (3.2 wt%). While both untreated lignin micro and nano-particles induce dramatic reduction of PLA thermal stability during TGA analysis, grafting SiP enables limiting this negative effect and enhancing the composites thermal stability that becomes very close to that of unfilled PLA. While the other lignin particles do not show any flame-retardant effect into PLA, the incorporation of LNP-SiP content at relatively low content (10 wt%) into PLA enables important increase of time to ignition as well as a reduction of pHRR. Remarkably enough, this effect is obtained even with the lowest LNP-SiP content (5 wt%). This study reports, for the first time, the interest of using lignin nanoparticles as flame-retardant agent in polymers. Results showed that grafting significant P content at the surface of lignin nanoparticles enables their use as flame-retardant additives, effective even at low incorporation content (5 wt%).

## Figures and Tables

**Table 1:** Phosphorus content determined by ICP of the different treated lignin particles.

Sample	Phosphorus Content (wt%)
LMP-diEtP	0.35 ± 0.02
LNP-diEtP	0.67 ± 0.02
LMP-SiP	0.1 ± 0.02
LNP-SiP	3.2 ± 0.02

**Table 2:** Number average molecular weight of processed PLA and PLA composites.

	Mn (g/mol)
PLA	80,000
10 wt% LMP	36,000
10 wt% LNP	39,000
10 wt% LMP-diEtP	33,000
10 wt% LNP-diEtP	55,000
10 wt% LMP-SiP	30,000
10 wt% LNP-SiP	40,000

**Table 3:** TGA results of neat PLA and related composites under nitrogen (10 °C/min).

	T_5%_ (°C)	T_10%_ (°C)	T_50%_ (°C)	Residue at 650 °C (%)
PLA	333	340	360	0.9
10% LMP	278	285	308	10.8
10% LNP	277	285	313	8.5
10% LMP-diEtP	263	278	294	9.7
10% LNP-diEtP	297	300	325	8.3
10% LMP-SiP	273	282	300	8.8
10% LNP-SiP	315	330	350	5.7

**Table 4:** Data of cone calorimeter test.

	TTI (s)	pHRR Variation (%)	Char (%)
PLA	68	/	0.1
10% LMP	65 ± 4	+12 ± 2	11.5 ± 0.4
10% LNP	34 ± 2	+5 ± 2	2 ± 0.1
10% LMP-diEtP	67 ± 2	+20 ± 8	8 ± 1.2
10% LNP-diEtP	54 ± 2	−6 ± 1	4.2 ± 1.3
10% LMP SiP	57 ± 4	+20 ± 5	7.3 ± 0.3
10% LNP SiP	86 ± 7	−18 ± 1	2 ± 4
5% LMP-SiP	57 ± 3	+ 18 ± 2	4.5 ± 0.2
5% LNP-SiP	84 ± 4	−11 ± 2	1 ± 0.1
